# Limbic areas are functionally decoupled and visual cortex takes a more central role during fear conditioning in humans

**DOI:** 10.1038/srep29220

**Published:** 2016-07-06

**Authors:** Chrysa Lithari, Stephan Moratti, Nathan Weisz

**Affiliations:** 1 Center for Cognitive Neuroscience, University of Salzburg, Austria; 2 Center for Mind/Brain Sciences, CIMeC, University of Trento, Italy; 3 Departamento de Psicología Básica I, Universidad Complutense de Madrid, Spain; 4 Laboratory for Cognitive and Computational Neuroscience, Universidad Politecnica de Madrid, Spain

## Abstract

Going beyond the focus on isolated brain regions (e.g. amygdala), recent neuroimaging studies on fear conditioning point to the relevance of a network of mutually interacting brain regions. In the present MEG study we used Graph Theory to uncover changes in the architecture of the brain functional network shaped by fear conditioning. Firstly, induced power analysis revealed differences in local cortical excitability (lower alpha and beta power) between CS+ and CS− localized to somatosensory cortex and insula. What is more striking however is that the graph theoretical measures unveiled a re-organization of brain functional connections, not evident using conventional power analysis. Subcortical fear-related structures exhibited reduced connectivity with temporal and frontal areas rendering the overall brain functional network more sparse during fear conditioning. At the same time, the calcarine took on a more central role in the network. Interestingly, the more the connectivity of limbic areas is reduced, the more central the role of the occipital cortex becomes. We speculated that both, the reduced coupling in some regions and the emerging centrality of others, contribute to the efficient processing of fear-relevant information during fear learning.

Learning the contingency between a threat signal and the potential danger it predicts is crucial for survival. Pavlovian fear conditioning is the most common laboratory model to study this particular type of learning: a previously neutral stimulus (CS+) is associated with an intrinsically aversive stimulus (US), while a second neutral stimulus remains unpaired. This association elicits a behavioral response in the presence of CS+ usually measured in terms of skin conductance, startle responses, pupil diameter or freezing behavior in animals^1^. Lesion studies in rats indicate a critical role of medial temporal structures, especially amygdala, in the acquisition of conditioned responses^2^. Studies in healthy humans implicate, apart from amygdala, an extended set of regions such as the pulvinar, thalamus^3^, anterior cingulate, insula and motor cortex (for a review see^4^). Simultaneous activation of these subcortical and cortical regions suggests that a fear relevant network rather than a distinct key region is responsible for fear perception.

Connectivity analysis is used to infer the coupling and decoupling between the nodes of a network. Indeed, an fMRI study showed increased functional connectivity between amygdala, object recognition areas (fusiform gyrus) and motor cortex in phobic participants when they passively watched phobic stimuli^5^ suggesting a shared neural network between fear conditioning and phobic reactions. Another fMRI study reported increased connectivity of right amygdala and visual cortex and decreased connectivity of left amygdala and occipito-temporal regions when participants were asked to identify fear faces^6^. These studies demonstrate that some regions exhibit fear-related coupling and others decoupling suggesting a re-routing of the functional pathways, which we intend to investigate in the present study. In a situation such as fear conditioning, an immediate response is demanded and this may in turn require a short-term re-organization of the brain functional connections as a response to learning but also in order to facilitate a rapid response. Using Graph Theory^7^ we aimed to uncover changes in the functional architecture of the brain network during fear conditioning. Graph theory offers tools to measure how efficiently information flows in a network, or how central the role of certain regions in the network is, features that are not accessible with standard connectivity analysis. With the excellent temporal resolution of MEG, we were able to describe fear-related network-level changes globally as well as locally in a time-frequency resolved manner. To our knowledge, there is no study using any neuroimaging modality that investigated fear conditioning under the framework of graph theory.

We implemented a typical fear-conditioning paradigm using flickering fearful faces at 15 Hz as CS and electrical stimulation at the left median nerve as the US, while MEG was recorded. We validated the effectiveness of the paradigm by means of startle responses^8^. In a companion paper^9^ we first reported in accordance with previous literature^8^ enhanced processing of CS+ not only on the visual cortex as expected, but also on subcortical structures. The current study expands the scope of the previous work, on induced responses with a main focus on characterizing connectivity patterns using graph theoretical tools in source space. The latter allows the investigation of fear-related differences in the organization of the brain functional network that are not evident with other analysis methods. Firstly we expected a higher excitability (lower alpha) of the somatosensory cortex during CS+ due to the expectancy of the painful US. Secondly, we hypothesized a distributed set of cortical as well as subcortical fear-relevant structures that compose the fear-network^4^ to emerge via our analysis using graph theoretical tools. More precisely, since prominent limbic-frontal connectivity is related to emotion regulation^10^,^11^, in a situation like fear conditioning, we expected decoupling phenomena in fear-relevant regions.

## Results

### Behavioral validation of conditioning

**Figure 1:**
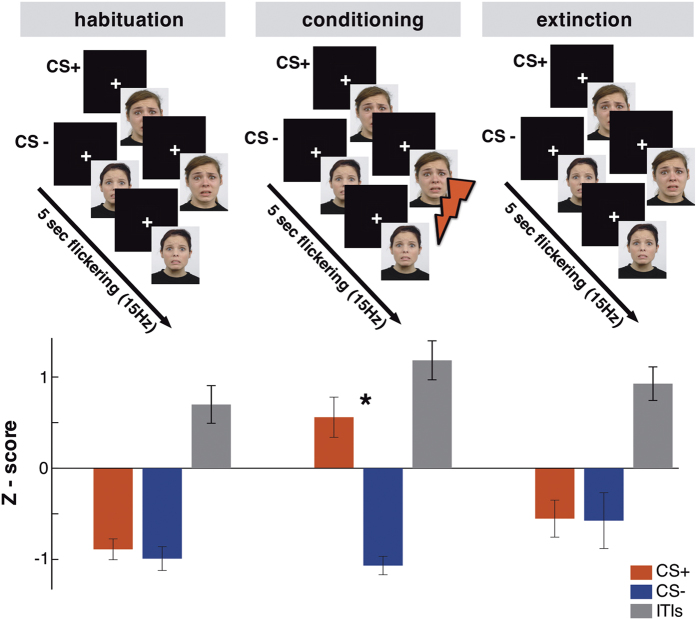
Top: the experimental design. Bottom: magnitude of the eye blink startle reflex expressed as z – scores. Startle reflex was modulated by the CS+ only during the conditioning phase (*p = 0.01). Note that the z – scores for CS were mostly negative because participants were more “responsive” to the white noise presented during ITI than during flickering CS (figure modified from^9^. The faces that appear in the figure belong to the Radboud Face Database^28^).

The startle responses of CS+ and CS− trials did not differ significantly during the habituation and extinction phases (Fig. 1, bottom). A reliable modulation was found in the conditioning phase where participants showed a higher startle response during CS+ compared to CS− (p = 0.01, t = 2.89, df = 17). The startle responses during the ITIs were significantly higher than both CS+ and CS− during habituation and extinction (p < 0.001; leading to the overall negative values), whereas during the conditioning phase they were at the same level with CS+. The differentiated startle response at CS+ validates the effectiveness of classical fear conditioning as it indicates a conditioning-specific activation of the defense system^12^.

### Decreased induced power

**Figure 2:**
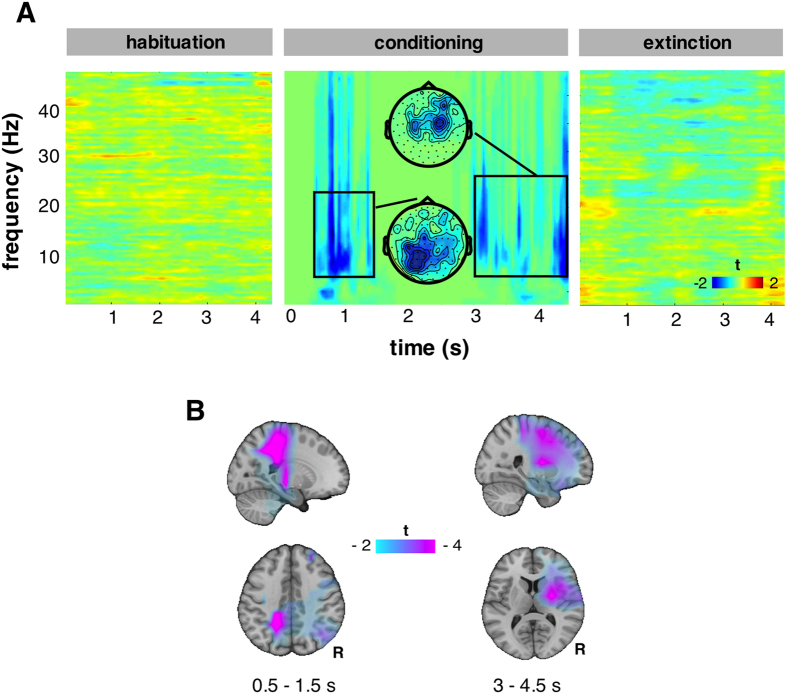
(**A**) CS+ vs. CS− statistics on time-frequency sensor space for the three experimental phases. Only during conditioning, we observed significant decreased induced power mainly in alpha and beta bands during CS+ in an early and a later point. (**B**) The two clusters were localized on source level using LCMV beamforming.

Statistical analysis CS+ vs. CS− revealed two significant clusters at sensor level: one at 3–4.5 s at central sensors, mostly contralateral to the expected US (*p* = 0.009) and a second one earlier at 0.5–1.5 s (*p* = 0.011) with posterior topography (Fig. 2A). The early effect is localized on left pre-central, post-central gyrus (*p* < 0.001) (Fig. 2B), which exhibited lower alpha and beta power during CS+ with respect to CS−. The same occurred on the right frontal inferior gyrus, right insula and putamen (*p* < 0.001) and to a lesser extent on dorsal frontal cortex (Fig. 2B) where the late effect was localized; prior to the upcoming noxious stimulus, less alpha and beta power was observed when compared to CS−. During habituation and extinction, we found no significant differences.

### Disconnection of subcortical structures

**Figure 3:**
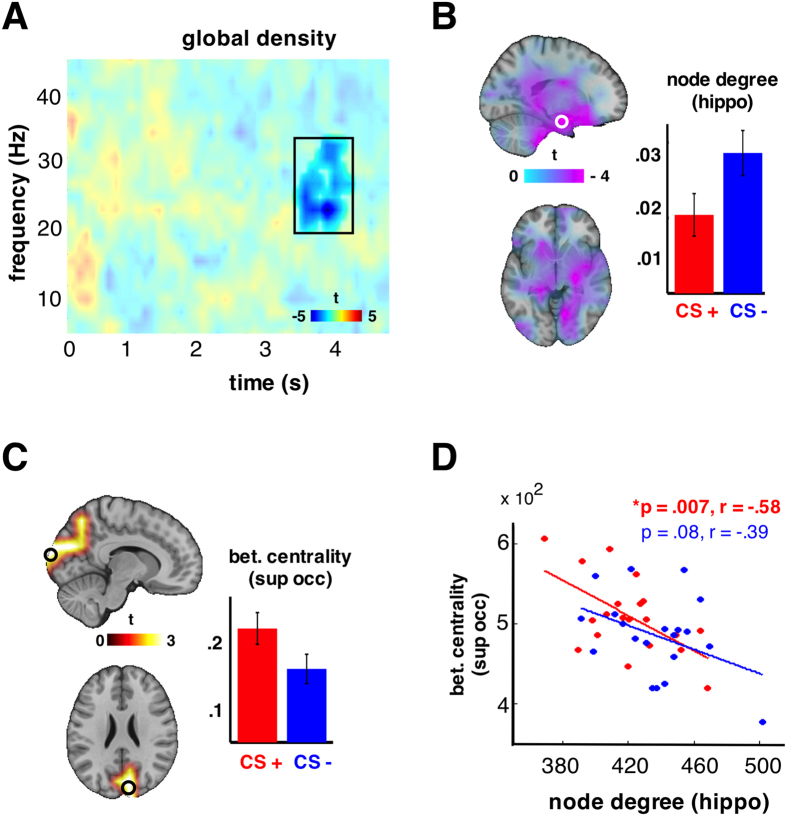
(**A**) the statistics (CS+ vs. CS−) on connection density are illustrated. A significant negative cluster (p = 0.013, corrected), indicating lower density during CS+ than CS−, was observed lasting from 3.1 till 4 sec and on the frequency range 18–28 Hz. The results in (**B–D**) refer to this time-frequency window of interest. (**B**) There was a significant reduction (p = 0.004, corrected) in the node degrees of subcortical structures bilaterally. The barplot depicts the node degree of left hippocampal gyrus (relative to the baseline) where the effect was maximized. Error bars correct for between-subject variability. (**C**) There was a significant increase (p = 0.013, corrected) on the betweenness centrality on occipital regions also bilaterally. The barplot depicts the betweenness centrality of left superior frontal gyrus (relative to the baseline) where the effect was maximized. Error bars correct for between-subject variability. (**D**) The effects described at (**B,C**) show a significant correlation only for CS+ (red dots, star indicates significant correlation). The less connections at hippocampal gyrus, the more shortest paths passing through occipital cortex.

Connection density, which indicates the number of functional connections in the brain, is decreased during CS+ with respect to CS− as shown by a significant negative cluster (*p* = 0.013) lasting from 3.2–3.8 s and 18–31 Hz (Fig. 3A). This means that during CS+ there are overall less functional connections in the brain network as compared to CS−. To obtain insights as to which regions contribute to this effect, we calculated the node degree of each voxel, which is the number of connections to other voxels, exactly at the time-frequency window of the connection density effect. A significant reduction of connections (*p* = 0.003) was found in several subcortical regions (insula, putamen, caudate, amygdala and hippocampal gyrus) bilaterally (Fig. 3B). That is, during CS+ these regions showed a relative disconnection with the rest of the functional network as compared to CS−. The effect was maximal (*t* = 4.2) at left hippocampus and amygdala (MNI [−20 −5 −20]). Following up this effect, we reported an increase of the local node degree relative to the baseline at left hippocampus for both CS+ and CS−, which is however lower for CS+ than for CS− (barplot in Fig. 3B). During habituation and extinction, we found no significant differences between CS+ and CS−.

### More shortest paths pass through occipital cortex

To further investigate the difference in the number of functional connections, we used the betweenness centrality, which is the number of shortest paths passing by a node, and is a measure of how central is the role of the node in a graph. Betweenness centrality was calculated for each voxel in the time-frequency window of interest where the connection density is significantly decreased due to fear conditioning. It is significantly increased (p = 0.013, corrected) in the superior and inferior occipital cortex and precuneus bilaterally (Fig. 3C) during CS+ as compared to CS−. That is, more connections that belong to shortest paths pass through these cortical regions during CS+ as compared to CS−. This effect is at its maximum at the right superior occipital gyrus (t = 3.72, MNI [10 −99.5 24.5]). Following up this effect with respect to the baseline, we reported an increase of the betweenness centrality at the superior occipital gyrus for both CS+ and CS−, which, however, is stronger for CS+ than for CS− (barplot in Fig. 3C). During habituation and extinction, we found no significant differences between CS+ and CS−. To sum up, during CS+ we observed a disconnection of subcortical regions and at the same time a re-direction of the shortest paths through occipital areas. Interestingly these two effects only significantly correlate for CS+ (p = 0.007, r = .58) (Fig. 3D). The fewer the connections at the left hippocampus (maximum node degree effect), the more the shortest paths passing from the superior occipital gyrus (maximum betweenness centrality effect).

### Limbic regions are functionally disconnected from fusiform and orbitofrontal gyri

**Figure 4:**
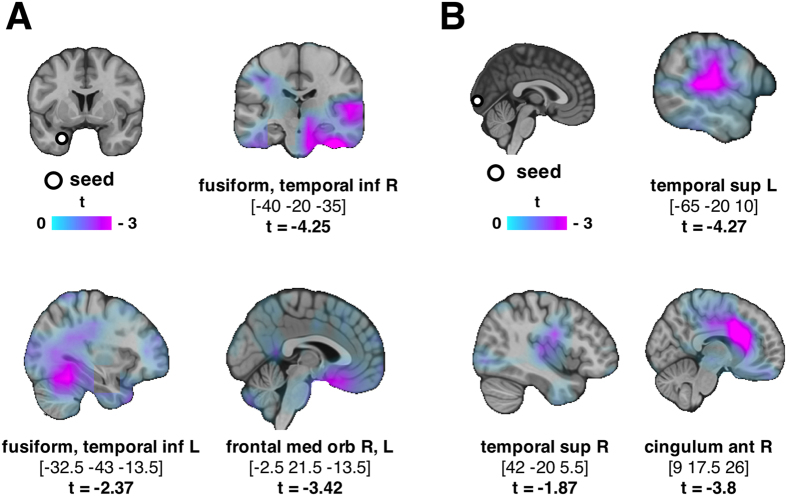
(**A**) A seed was placed at hippocampal gyrus (MNI [−20 −5 −20]) where the node degree effect was most significant. Connectivity between the seed and fusiform, temporal and frontal regions was lower during CS+ with respect to CS− (p < 0.05, not corrected). (**B**) A seed was placed at occipital gyrus (MNI [10 −99.5 24.5]) where the betweenness effect was most significant. Temporal cortex and anterior cingulum exhibited lower functional connectivity with the seed during CS+ with respect to CS− (p < 0.05, not corrected).

We showed that during CS+, the node degree in a diverse set of subcortical regions is lower, that is, these regions have fewer connections, than during CS−. Less connections, in other words less “1“s in the binary adjacency matrix, implies lower connectivity values (imaginary coherence) before the thresholding. To identify which regions lost their connectivity to these deep structures, we placed a seed on the left hippocampal gyrus, where the node degree effect has its maximum (t = −4.2, MNI [−20 −5 −20]) and we examined if the connectivity of all voxels to/from the seed differed between CS+ and CS−. A set of areas exhibited reduced connectivity with the seed in the time-frequency window of interest determined by the significant cluster on connection density. When statistics are corrected (montecarlo), it is only the right fusiform that showed reduced connectivity with the seed, however, the uncorrected values were plotted to show the complete image of reduced short and long-range connectivity. As shown in Fig. 4A, deep limbic areas are disconnected from both nearby regions (left fusiform), and long-distant areas (right temporal, fusiform and orbitofrontal gyrus). The maximum of this effect is at the right fusiform (t = −4.27, MNI [−40 −20 −35]). During habituation and extinction, there were no significant differences.

### Occipital lobe is functionally disconnected from temporal gyri and anterior cingulate

We observed that during CS+, the betweenness centrality of occipital cortex is significantly higher, giving to this region a more central role, as compared to CS−. This implies the probability that the connectivity of the occipital cortex with the rest of the brain might have been altered due to fear conditioning. Indeed, placing a seed there and looking at differences in the connectivity values between CS+ and CS−, revealed lower connectivity with temporal lobes and anterior cingulate bilaterally (Fig. 4B). The maximum of this effect is at left temporal superior cortex (t = −4.27, MNI [−65 −20 10]). During habituation and extinction, there were no significant differences.

## Discussion

In considering the brain functional network as a complex graph that is dynamically modified in response to, but also in anticipation of, environmental changes, we intended to examine how fear conditioning alters the graph structure and properties. We recorded MEG during a classical fear-conditioning paradigm and we focused our analysis firstly on induced power, but mainly on brain functional connectivity using graph theory. Confirming our first hypothesis, fear conditioning caused a decrease in power in alpha and beta bands prior to the upcoming aversive US and this effect was localized on the somatosensory cortex and insula. The most interesting finding however is the tendency of the brain functional network to ‘lose’ some of its functional connections during CS+ with respect to CS−. Thus, our second hypothesis about decreased connectivity at fear-relevant regions is also verified, since it is subcortical limbic structures that lose their connections. At the same time a higher number of shortest-paths are routed through occipital gyrus, which takes a more central role in the brain functional network. These two phenomena are correlated: the more connections lost at hippocampus, the more central the role of the occipital cortex.

Reduced power in alpha band is an index of the local excitability of relevant cortices prior to a stimulus^13^, in our case, an expected painful somatosensory one. The early (0.5–1.5 s) decrease of alpha and beta power on somatosensory cortex reflects top-down biases that modulate excitability^13^ of somatosensory cortex. Later on (3–4.5 s), also insula is involved exhibiting decreased alpha and beta power. This effect could not be uncovered by the conventional evoked responses analysis that pointed to generalized effects related to the *enhanced cortical processing* of CS+, in this case the visual cortex^9^. However, the process of averaging for evoked responses penalizes single-trial modulations of brain activity that are not precisely time-locked to the stimulus. Indeed, analysis of induced responses on source level suggested increased excitability (reduced alpha and beta power) on the cortices relevant to the modality of the aversive US, in this case the somatosensory cortex. This could be interpreted as effects related to the *expectancy* of a stimulus on the relevant cortices as previously suggested by Langner and colleagues^13^. Later on, closer to the upcoming US, increased excitability was also found at insula, a structure activated in fear conditioning^14^ and during anticipation of painful stimuli in particular^15^. Taken together, we reported increased excitability at the cortex relevant to the modality of the upcoming US, but also at a structure involved in the processing of aversive stimuli independently of the modality of the expected US.

We found a reduction of connections in low gamma band caused by fear conditioning. Fear conditioning is a situation that involves emotional learning and requires a rapid response and a re-organization of the brain functional network seems critical. This re-organization evoked by fear is apparently a large-scale one that it is even reflected in terms of density, one value that describes a graph globally.

The general decrease of the number of connections was localized on subcortical structures including limbic areas (insula, putamen, amygdala, hippocampus). The communication-though-coherence hypothesis, assumes that the absence of functional connections (coherence) prevents communication between neuronal populations^16^. That is, in our case, subcortical brain structures loose some connections with the rest of the brain. The connectivity of amygdala is altered in a neighboring region even during rest following fear conditioning^17^. Stronger connectivity between amygdala and prefrontal cortex predicts effective emotion regulation and lower levels of anxiety^10^,^11^. Fear conditioning, even though in the laboratory, is a stressful situation where an immediate response is required. In this context, the reduction in density, and thus connectivity, is in line with previous findings and can be interpreted as a quick adaptation of the brain functional network to a situation far from emotion regulation, probably “sacrificing” some non-fundamental connections to increase the readiness to respond.

Indeed, placing a seed in the left hippocampus, revealed a functional disconnection with the fusiform and orbitofrontal gyri. The latter is preferentially involved in emotional as compared to cognitive perspective taking^18^ and it is anatomically connected with the limbic system in monkeys^19^. The left fusiform gyrus on the other hand is suggested as the visual word form area^20^ and as such, a disconnection from limbic areas during non-verbal stimuli processing is meaningful. Given the fact that this effect is distributed all over the brain, no significant spatial cluster was formed. However, our results do support the disconnection of the limbic areas from both short and long distance structures. One might speculate that the disconnection of limbic areas from the rest of the brain has, at least to some extent, a protecting role towards interference: via this architecture, the subcortical flow of information is locally integrated/reinforced and, at a later step, spread throughout the brain network.

To sum up till this point, fear conditioning caused a disconnection of fear-relevant subcortical regions that also significantly affected the connection density of the brain functional network. Our finding is in line with the notion of a fear relevant network^4^,^6^ and we reported for the first time a specific disconnection of subcortical regions elicited by fear conditioning.

To explore the degree effect deeper, we also investigated the betweenness centrality of each node, that is, if fear conditioning affected the number of the shortest paths that pass through each node. Indeed we found a significant increase of betweenness centrality in cortical areas such as the superior and inferior occipital gyri and precuneus bilaterally during CS+ with respect to CS−. Nodes with high betweenness centrality participate in many shortest paths and consequently act as important points of control of information flow^21^, the so-called *hubs* of a network. The deprecation of connections at subcortical regions can be interpreted together and in line with a need for a re-direction of the existing ones, that is, leading to a re-direction of the shortest paths as well. Indeed, the negative correlation of these effects supports such an interpretation: the more connections “lost” at subcortical structures (hippocampus) as a response to fear-conditioning, the more shortest paths are re-directed through the occipital cortex. Given the nature of the CS, one can speculate that the locus of CS processing, that is the occipital lobe, plays a more central role, during fear conditioning. It remains to be investigated if the modality of the CS is indicative of re-routing information in the brain functional network.

Due to the correlative nature of the effects, we cannot make an assumption regarding which of the two effects caused the other. An alternative interpretation could be that the re-direction of the shortest paths to pass through visual processing regions led to reducing some subcortical connections in order to keep the communication cost low. Whatever their exact relation, the hub-like function of occipital gyrus together with a decreased number of subcortical connections seems to facilitate fear learning, a situation that, under a common evolutionary framework, requires an immediate response. However, given the graph-theoretical definition of the hub (betweenness centrality higher than the mean of all nodes plus one standard deviation^22^), we cannot deduce that the occipital cortex acts as one, since what we compare herein is the betweenness centrality CS+ vs. CS− and not the betweenness centrality of one node vs. the rest of the nodes.

Investigating further the betweenness effect, we placed a seed in the occipital gyrus, where it was maximized and we noted decreased connectivity with temporal lobes and cingulate bilaterally. Superior temporal gyrus is involved in the perception of emotions in facial stimuli (CS were fearful faces)^23^, while the anterior cingulate plays a central role in pain^24^. Considering our findings under the framework of graph theory, it is interesting to mention that the more central role given to the occipital cortex (by routing more shortest paths through it) is accompanied with a loss of connections between occipital cortex and other areas. Our findings again point to the fact that in a critical and stressful situation such as fear, *disconnection* might be a mechanism that facilitates processing of aversive stimuli and it makes sense that the connections to be “cut” should be the non-fundamental ones.

One could raise a concern on the method we used to threshold the connectivity values to obtain the adjacency matrices. Thresholding is a widely discussed issue when applying graph theory on brain networks^25^,^26^. A range of arbitrary thresholds or a range of equal, yet arbitrary, densities is common practice. Equalizing the connection density of the graph is common in order to look at organizational differences usually between patients and healthy participants^27^. However, applying this approach in within subjects contrast assumes that different brain states (herein fear conditioning) do not have any impact on the connectivity values, and thus on the connection density. The stimulation we used lasted 4 sec and could potentially have an effect during any moment and in any frequency band, thus, any change in the connection density would be worthy to investigate. Indeed, we observed a difference in the connection density only close to the upcoming US in low gamma band and only during conditioning and not during habituation and extinction. The node degree analysis was performed to follow up this global effect. The betweenness centrality analysis, also driven by the global effect on connection density, differed significantly in a different location (visual cortex) than the node degree (subcortical areas) in the same time-frequency window. Yet, we only considered the effects that were significant across all the threshold range we tested. Nevertheless, considering the influence of connection density to the more complex graph theory measures we performed additional analysis matching the connection density for CS+ and CS− and normalizing the node betweenness centrality of our obtained graphs with that of random graphs of same connection density. We then contrasted the betweenness centrality of each node between CS+ and CS− for connection densities equal to 0.5, 0.6 and 0.7 and indeed it is higher during CS+ than during CS− mainly at the visual cortex (as described in our results) but also at other cortical areas (Supplementary Fig. S1). We are confident, thus, to report a re-organization of the brain functional network due to fear conditioning and not just a spurious reduction in the connection density.

Of note, we have utilized fearful faces as CS. This might have introduced specific effects of fearful face processing. However, as both the CS− and CS+ were fearful faces, these effects have been controlled for.

In conclusion, we hypothesized that human brain during fear conditioning apart from local modulations of cortical power, responds also with a re-organization of its functional connections, a phenomenon observable only using graph theoretical measures. The fear-relevant structures were indeed de-coupled from the rest of the network, while the cortex processing the fear-related information, in our case visual, took a more central role. Importantly, these two effects were correlated, probably to the direction of efficient fear learning.

## Methods

### Participants

Twenty right-handed participants (10 females; age: 28.05 ± 3.3 years) with normal or corrected-to-normal vision, no neurological or psychiatric disorders and no family history of photic epilepsy took part in the experiment. The Ethical Committee of the University of Trento approved the experimental protocol and the experiments were performed in accordance with the approved guidelines. Participants confirmed that they understood the experimental procedure and gave their written informed consent.

### Stimuli and procedure

Two fearful faces of Caucasian adult women from the Radboud Faces Database^28^ were used as CS+ and CS− counterbalanced among participants. The CS was flickering at 15 Hz for 5 sec on a black screen with a refresh rate of 60 Hz (Fig. 1, top; 2 frames “on” and 2 frames “off”). The stimuli were projected on a screen inside an MEG shielded room through a video projector (Panasonic PT-D7700E) and a mirror system. A white fixation cross was presented on the screen during the inter-trial-interval (ITI), which jittered between 7 and 8 sec. The precise start and end of each trial was determined by a photodiode sensitive to luminance change placed on the screen inside the MEG room.

The US was a 100 ms electric pulse stimulating the participant on the left median nerve. Two electrodes (cathode proximal) were connected directly to a galvanically insulated electrical stimulator. A step-wise procedure was followed before the experiment to define the individual pain threshold. Participants were asked to rate the intensity of the pulse using a scale of 0 (not perceived) to 7 (very painful). It was explained to them that the intensity used during the experiment should be tolerable, however it should be sufficiently unpleasant in order to be salient. The target rated pain level for each individual was 6 and this procedure led to an average of 33.05 ± 16.3 mA at 200 Volts. The delivery of the US jittered between 4600 and 4800 ms after the CS+ start and they terminated simultaneously.

The experiment consisted of three phases: habituation, conditioning and extinction. During habituation, 18 CS+ and 18 CS− trials were presented in random order in two blocks each lasting 6 minutes, but the CS+ was never paired with an US. During the conditioning phase, 40 CS+ and 40 CS− trials were presented, while CS+ trials were paired with an US. Conditioning included five blocks while the extinction phase was identical to the habituation. The whole experiment lasted for approximately 1 hour. Participants were instructed to pay attention, as they would be asked to report the stimulus predicting the delivery of the painful stimulus at the end of each block^29^,^30^. All participants reported that they were aware of the CS−US contingency already after the first block of conditioning.

### Startle responses

As a measure of the effect of conditioning on the activation of the defense system^12^, we recorded participants’ startle responses elicited by a short (100 ms) white noise presented binaurally in selected trials through a pneumatic tube system. Startle responses were extracted from the vertical Electro-occulo-graphic (EOG) bipolar recordings. For each of the three phases of the experiment 33 startle responses were recorded; 11 in CS+, 11 in CS− and 11 during ITIs. Artifact contaminated trials were detected separately for each participant and excluded from the analysis. The EOG data from two participants were excluded because of recording problems. The magnitude of the startle response was calculated by subtracting the peak amplitude during 20–120 ms post-stimulus from a 200 ms prestimulus period. The absolute values were then expressed in z-scores to account for individual variability^8^,^31^. Repeated-measures ANOVA was conducted with phases (habituation, conditioning, extinction) and condition (CS+, CS−, ITI) as within subject factors.

### MEG recording and preprocessing

MEG was recorded at a sampling rate of 1 kHz using a 306-channel (204 first order planar gradiometers, 102 magnetometers) VectorView MEG system (Elekta-Neuromag Ltd., Helsinki, Finland) in a magnetically shielded room (AK3B, Vakuum Schmelze, Hanau, Germany). The head positions of the individuals relative to the MEG sensors were continuously controlled within a block using three coils placed at three fiducial points (nasion, left and right preauricular points). Head movements did not exceed 1.5 cm within and between blocks.

Data were treated offline using the Fieldtrip toolbox^32^. CS+ and CS− trials of 2 sec pre- and 6 sec post-stimulus were extracted from the continuous data stream based on the photodiode signal. Trials containing physiological or acquisition artifacts were visually inspected and rejected. The number of CS+ and CS− trials were equalized for each subject within each of the experimental phases to ensure that our results were not confounded by systematic differences in signal-to-noise ratio.

### Analysis of induced responses at sensor level

Induced responses to CS were first analyzed on a sensor level. Spectral analysis was performed on single trials (Hanning tapering; 2–45 Hz in 1 Hz steps; time windows of 5 cycles per frequency; −1–5.5 s; sliding in 50 ms steps). Horizontal and vertical planar gradients of the magnetic field at each gradiometer were analyzed separately. The sum of both directions (combined planar gradient) was computed to obtain the power at each sensor irrespective of the orientation of the gradients^33^. We validated induced power differences between CS+ and CS− conditions in all experimental phases (0–4.5 s, 2–45 Hz).

### Analysis of induced responses at source level

A structural MRI (4T Bruker MedSpec, Siemens) was available for 15 out of 20 participants. Three anatomical landmarks (nasion and left/right pre auricular points) and the head shape were digitized with a Fastrak 3D digitizer (Polhemus, Colchester, VT, USA) and co-registered on the individual segmented MRIs. Co-registered MRIs were segmented using SPM7 to derive the outer brain surface allowing the calculation of a semi-realistic head model^34^. For those participants with no structural MRI, an MNI template brain was warped (affine transformation) to minimize the difference to the individually digitized head shape. An equally spaced grid (1.5 cm resolution) was fitted to a brain volume obtained from a segmented template MNI brain. This template grid was subsequently warped into the individual headspace ensuring the same amount of grid points at the same brain location in MNI space across participants^35^. The grid positions in individual head coordinates, the sensor positions relative to the head and the head model were used to calculate the leadfield. Both magnetometers and gradiometers were included in the source estimation after appropriate adjustment of the balancing matrix based on the distance of the gradiometers (17 mm). To estimate the generators of the sensor level effects, single trial sensor level time series were multiplied with a spatial filter derived from a Linearly Constrained Minimum Variance (LCMV) beamformer^36^ (−1–5.5 s). Time-frequency analysis was then performed on source level signals using Hanning tapers (2–45 Hz in 1 Hz steps; time windows of 5 cycles per frequency; −1–5.5 s sliding in 50 ms steps).

### Functional connectivity and graph theory

A Fast Fourier Transform (Hanning tapers; 2–45 Hz in 1 Hz steps; time windows of 5 cycles per frequency; 0–4.5 s sliding in 50 ms steps) was applied to the virtual sensors as described above. The imaginary part of coherence^37^ was calculated for all possible pairs of voxels for 2–45 Hz resulting in time-frequency resolved all-to-all connectivity values. To obtain a binary adjacency matrix (zeros indicating absence, ones indicating presence of a functional connection) for graph theoretical analysis, the all-to-all connectivity matrix needs to be thresholded. There is no objective way to decide the threshold value in graph theoretical approaches^16^. A range of reasonable thresholds (.04, 0.05, 0.06, 0.07) was applied on coherence values to pass from weighted to binary graphs and effects were statistically tested across that range (see next section). Thresholding yielded an adjacency matrix (889 × 889) for each point in time-frequency space. Global and local graph theoretical measures were calculated on the adjacency matrices. We obtained the connection density for each time-frequency bin and node degrees and betweenness centrality for each voxel and for each time-frequency bin.

The degree of a node is the number of edges connected to the node; likewise betweenness centrality is defined as the fraction of all shortest paths in the network passing through that node^38^. Both measures assess the importance of nodes within a network, possibly identifying hubs, that is, brain regions that facilitate functional integration and interact with many other regions^38^. Given the significant effects for node degree and betweenness centrality, we examined their relationship using Pearson correlation. As a last step, we studied seeded connectivity, using as seed regions the regions where the previous effects were maximized, that is a seed was placed on the hippocampus where the node degree effect was maximal and on the occipital lobe, where the betweenness effect was maximized and we compared connectivity of the seed to all other grid points between CS+ and CS−.

### Statistics

All CS+ vs. CS− tests were performed during habituation, conditioning and extinction phases, but significant differences were expected only during conditioning. On a sensor level, a dependent samples t-test was carried out on time-frequency data to test for differences during habituation, conditioning and extinction periods. To control for multiple comparisons, a non-parametric Monte-Carlo randomization test was undertaken^39^. The t-test was repeated 1000 times on data shuffled across conditions and the largest t-value of a cluster coherent in time and space was retained. The observed clusters were compared against the distribution obtained from the randomization procedure and were considered significant when their probability was below 5%. The connection density was also tested in the same way looking for significant clusters in time-frequency space.

At a source level, induced power effects were tested with a dependent samples t-test (Monte-Carlo correction) in the time-frequency windows derived from the sensor level statistics. Significant clusters were identified in space. The same approach was followed for the local graph measures on each voxel (node degree, betweenness centrality) on the time-frequency window of interest derived from significant effects on connection density. In respect of the graph theory measures, statistics were performed for the whole range of thresholds (.04, 0.05, 0.06, 0.07) and only effects that survived across all thresholds are presented in the paper. For illustration purposes we used a threshold of 0.05.

## Additional Information

**How to cite this article**: Lithari, C. *et al*. Limbic areas are functionally decoupled and visual cortex takes a more central role during fear conditioning in humans. *Sci. Rep.* **6**, 29220; doi: 10.1038/srep29220 (2016).

## Supplementary Material

Supplementary Information

## References

[b1] LeDoux J. E. The Emotional Brain. (Simon & Schuster, New York, 1996).

[b2] LaBar K. S. & LeDoux J. E. Partial disruption of fear conditioning in rats with unilateral amygdala damage. Behav. Neurosci. 110, 991–997 (1996). 8919001 10.1037//0735-7044.110.5.991

[b3] Morris J. S., Öhman A. & Dolan R. J. A subcortical pathway to the right amygdala mediating “unseen“ fear. Proc. Nat. Acad. Sci. 96, 1680–1685 (1999). 9990084 10.1073/pnas.96.4.1680 PMC15559

[b4] Büchel C. & Dolan R. J. Classical fear conditioning in functional neuroimaging. Curr Opin Neurobiol. 10, 219–223 (2000). 10753800 10.1016/s0959-4388(00)00078-7

[b5] Ahs . Disentangling the web of fear: amygdala reactivity and functional connectivity in spider and snake phobia. Psychiatry Res. 15, 103–108 (2009). 10.1016/j.pscychresns.2008.11.004 19321315

[b6] Das . Pathways for fear perception: modulation of amygdala activity by thalamo-cortical systems. NeuroImage 26, 141–148 (2005). 15862214 10.1016/j.neuroimage.2005.01.049

[b7] Sporns O. Graph theory methods for the analysis of neural connectivity patterns. Neuroscience Databases, A Practical Guide, 171–185, (Springer: US, 2003).

[b8] Stolarova M., Keil A. & Moratti S. Modulation of the C1 visual event-related component by conditioned stimuli: evidence for sensory plasticity in early affective perception. Cereb. Cortex 16, 876–887 (2006). 16151178 10.1093/cercor/bhj031

[b9] Lithari C., Moratti S. & Weisz N. Thalamocortical interactions underlying visual fear conditioning in humans. Hum. Brain Map. 36, 4592–4603 (2015). 10.1002/hbm.22940 PMC6869800 26287369

[b10] Kim M. J. . The structural and functional connectivity of the amygdala: From normal emotion to pathological anxiety. Beh Brain Res 223, 403–410 (2011). 10.1016/j.bbr.2011.04.025 PMC3119771 21536077

[b11] Banks S. J., Eddy K. T., Angstadt M., Nathan P. J. & Phan K. L. Amygdala-frontal connectivity during emotion regulation. Social Cognitive & Affective Neurosci 2, 303–312 (2007). 10.1093/scan/nsm029 PMC2566753 18985136

[b12] Lang P. J., Bradley M. M. & Cuthbert B. N. Emotion, attention and the startle reflex. Physiol. Rev. 97, 377–395 (1990). 2200076

[b13] Langner R. . Modality-specific perceptual expactations selectively modulate baseline activity in auditory, somatosensory and visual cortices. Cereb. Cortex 21, 2850–2862 (2011). 21527785 10.1093/cercor/bhr083

[b14] Büchel C., Morris J., Dolan J. M. & Friston K. J. Brain systems mediating aversive conditioning: an event-related fMRI study. Neuron 20, 947–957 (1998). 9620699 10.1016/s0896-6273(00)80476-6

[b15] Ploghaus A. . Dissociating pain from its anticipation in the human brain. Science 284, 1979–1981 (1999). 10373114 10.1126/science.284.5422.1979

[b16] Fries P. A mechanism for cognitive dynamics: neuronal communication through neuronal coherence. Trends Cogn. Sci. 9, 474–480 (2005). 16150631 10.1016/j.tics.2005.08.011

[b17] Schultz D. H., Balderston N. L. & Helmstetter F. J. Resting-state connectivity of the amygdala is altered following Pavlovian fear conditioning. Front Hum Neurosci 6, 242 (2012). 22936906 10.3389/fnhum.2012.00242 PMC3426015

[b18] Hynes C. A., Baird A. A. & Grafton S. C. Differential role of the orbital frontal lobe in emotional versus cognitive perspective-taking. Neuropsychologia 44, 374–383 (2005). 16112148 10.1016/j.neuropsychologia.2005.06.011

[b19] Porrino L. J., Crane A. M. & Goldman-Rakic P. S. Direct and indirect pathways from the amygdala to the frontal lobe in rhesus monkeys. J. Comp. Neurol. 198, 121–136 (1981). 6164704 10.1002/cne.901980111

[b20] McCandliss B. D., Cohen L. & Dehane S. The visual word form area: expertise for reading in the fusiform gyrus. Trends Cogn. Sci. 7, 293–299 (2003). 12860187 10.1016/s1364-6613(03)00134-7

[b21] Freeman L. C. A set of measures of centrality based on betweenness. Sociometry 40, 35–41 (1977).

[b22] Sporns O. Honey C. J. & Kötter R. Identification and classification of hubs in brain networks. PLOS One, doi: 10.1371/journal.pone.0001049 (2007). PMC2013941 17940613

[b23] Radua J. . Neural responses to specific components of fearful faces in healthy and schizophrenic adults. NeuroImage 49, 939–946 (2010). 19699306 10.1016/j.neuroimage.2009.08.030

[b24] Johansen J. P., Fields H. L. & Manning B. H. The affective component of pain in rodents: direct evidence for contribution of the anterior cingulate cortex. Proc. Nat. Acad. Sci. USA 98, 8077–8082 (2001). 11416168 10.1073/pnas.141218998 PMC35470

[b25] van Wijk B. C. M., Stam C. J. & Daffertshofer A. Comparing brain networks of different size and connectivity density using graph theory. PLOS ONE doi: 10.1371 (2010). 10.1371/journal.pone.0013701 PMC2965659 21060892

[b26] De Vico Fallani F., Ricchiardi J., Chavez M. & Achard S. Graph analysis of functional brain networks: practical issues in translational neuroscience. Philos Trans R Soc B Biol Sci. 369, 20130521 (2014). 10.1098/rstb.2013.0521 PMC4150298 25180301

[b27] De Vico Fallani F. . Cortical functional connectivity networks in normal and spinal cord injured patients: Evaluation by graph analysis. Hum Brain Mapp 28, 1334–1346 (2007). 17315225 10.1002/hbm.20353 PMC6871447

[b28] Langner O., Dotsch R., Bijlstra G., Wigboldus D. H. J., Hawk S. T. & van Knippenberg A. Presentation and validation of the Rabdoud Faces Database. Cogn. and Emotion 24, 1377–1388 (2010).

[b29] Knight D. C., Smith C. N., Cheng D. T., Stein E. A. & Helmstetter F. J. Amygdala and hippocampal activity during acquisition and extinction of human fear conditioning. Cogn. Affect. Behav. Neurosci. 4, 317–325 (2004). 15535167 10.3758/cabn.4.3.317

[b30] Miskovic V. & Keil A. Acquired fears reflected in cortical sensory processing: a review of electrophysiological studies of human classical conditioning. Psychophysiology 49, 1230–1241 (2012). 22891639 10.1111/j.1469-8986.2012.01398.x PMC3422776

[b31] Keil A., Stolarova M., Moratti S. & Ray W. J. Adaptation in human visual cortex as a mechanism for rapid discrimination of aversive stimuli. NeuroImage 36, 472–479 (2007). 17451974 10.1016/j.neuroimage.2007.02.048 PMC2034335

[b32] Oostenveld R., Fries P., Maris E. & Schoffelen J. M. FieldTrip: Open source software for advanced analysis of MEG, EEG and invasive electrophysiological data. Comp. Intell. Neurosci. dx.doi.org/10.1155/2011/156869 (2011). 10.1155/2011/156869 PMC3021840 21253357

[b33] Medendorp W. P. . Oscillatory activity in human parietal and occipital cortex shows hemispheric lateralization and memory effects in a delayed double-step saccade task. Cereb. Cortex 17, 2364–2374 (2007). 17190968 10.1093/cercor/bhl145

[b34] Nolte G. The magnetic lead field theorem in the quasi-static approximation and ist use for magnetoencephalography forward calculation in realistic volume conductors. Phys. Med. Biol. 48, 3637–3652 (2003). 14680264 10.1088/0031-9155/48/22/002

[b35] Larson-Prior L. J. . Adding dynamics to the Human Connectome Project with MEG. NeuroImage 80, 190–201 (2013). 23702419 10.1016/j.neuroimage.2013.05.056 PMC3784249

[b36] van Veen B. D., van Drongelen W., Yuchtman M. & Suzuki A. Localization of brain electrical activity via linearly constrained minimum variance spatial filtering. IEEE Trans. Biomed. Eng. 44, 867–880 (1997). 9282479 10.1109/10.623056

[b37] Nolte G. . Identifying true brain interaction from EEG data using the imaginary part of coherency. Clin. Neurophysiol. 115, 2292–2307 (2004). 15351371 10.1016/j.clinph.2004.04.029

[b38] Rubinov M. & Sporns O. Complex measures of brain connectivity: Uses and interpretations. NeuroImage 53, 1059–1069 (2010). 19819337 10.1016/j.neuroimage.2009.10.003

[b39] Maris E. & Oostenveld R. Nonparametric statistical testing of EEG and MEG data. J. Neurosci. Methods 164, 177–190 (2007). 17517438 10.1016/j.jneumeth.2007.03.024

