# Supplementary material for: Limbic areas are functionally decoupled and visual cortex takes a more central role during fear conditioning in humans
Source: Sci Rep. 2016 Jul 6;6:29220. doi: 10.1038/srep29220 (PMC4933895; doi:10.1038/srep29220)
Supplement: Supplementary Information [file srep29220-s1.pdf]

Title:

Limbic areas are functionally decoupled and visual cortex takes a more central role during fear conditioning in humans.

List of authors:

Chrysa Lithari*, Stephan Moratti, Nathan Weisz

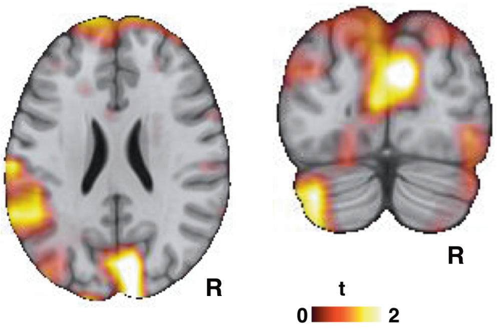

Supplementary Figure 1: Contrast of betweenness centrality CS+ vs. CS- (masked p < .05, uncorrected) revealed higher betweenness centrality during CS+ with respect to CS- at the visual cortex and other cortical areas, supporting our initial analysis presented in the manuscript. The connection density was matched between conditions equal to 0.7 and the betweenness centrality was normalized with that of random graphs of same connection density. Similar results were obtained also for matched connection density of 0.5 and 0.6.
